# Prosecution for Mal-Practice

**Published:** 1849-07

**Authors:** 


*Boston Medical and Surgical Journal.*

---

*Prosecution for Mal-Practice.*—By Walter K. Manning, Esq.—[Communicated for the Boston Medical and Surgical Journal.]

Supreme Court at Lowell.—Francis Conant *vs.* Peter Manning. This was an action against Dr. Manning, of Lunenburg, Mass., a surgeon of very respectable standing, for an alleged want of skill and care in the reduction and cure of a dislocation of the plaintiff's thigh. It appeared in evidence that on the 27th of December, 1847, Mr. Conant, who lives in Stow, was driving an ox team with a load of wood upon a sled, in the town of Lunenburg, and fell under his oxen, and the load passed over him. He was severely bruised; and it was found that his lower jaw was fractured on each side, and his thigh-bone dislocated; that Dr. Manning was called immediately, and after great exertion, for several hours, the limb was reduced, and the usual measures were adopted to retain the limb in its place. But it was ascertained that the dislocation was accompanied with a very considerable fracture of the socket, and that it was extremely difficult to keep the head of the bone in its place. Mr. C continued upon his bed three or four weeks, and then began to move about the house on his crutches. He then became very impatient, and was desirous of returning home; but being admonished by the Doctor, of the danger of leaving, he was induced to remain until the 15th of February, 1848, when, against the advice of Dr. M., he left and returned home. On the 2d of March, he returned to Lunenburg to see the Dr., assuring him that his limb was doing well, and that he was much better, and indeed had found the belt prescribed by the Dr. unnecessary, and had laid it aside. Dr. M. then examined him, and found that his limbs were nearly of the same length, and that he had considerable use of the limb that had been dislocated. He returned home, leaving the impression upon the mind of the Doctor, and all who saw him, that he was doing as well as could be expected under the circumstances, though Dr. M. urged upon him the importance of resuming the belt.

On the 22d of March, having been advised by his family physician to apply for aid at the Massachusetts General Hospital, after an examination by the surgeons of that institution Mr. C. was subjected to a severe operation for the purpose of improving the condition of his limb. The operation was in a measure successful; he had a better use of his limb than before. But an entire restoration has not been attained, nor was it expected by the surgeons at the hospital; the limb remaining more than two inches shorter than before the injury.

The plaintiff introduced witnesses who testified, that while he remained under the care of Dr. M., the latter told him frequently that he "was getting along well," that all was right, &c. His counsel contended that the surgeon had misrepresented his condition, and that he had thereby suffered damage. Several surgeons of the hospital were called as witnesses, who testified that the case of Mr. C. was most difficult—both to understand and to manage; that it was not to be expected that under any treatment the limb could be restored, and that there was nothing shown in the case that indicated either want of skill or want of care in Dr. Manning. One of those gentlemen who had been present and heard the whole testimony, and who had been notified that his opinion would be called for, gave his opinion that there appeared to be no fault on the part of Dr. Manning, but that the case had been treated by him as well and as successfully as it could have been treated under the circumstances. It was admitted by the counsel for the plaintiff, that there was no fault or want of skill in reducing and curing the fracture of the jaw, nor in reducing or subsequent treatment of the hip, but it was contended that the Dr. had misrepresented the condition of his patient. The jury returned a verdict for the plaintiff for

$362,50!!!! The defendant being dissatisfied with the result, made a motion at once for a new trial, on the ground that the verdict was manifestly against the weight of evidence, and moved the judge who held the court (Mr. Justice Dewey), that the evidence might be reported and submitted to the whole court for that purpose, which motion was sustained by the court.

E. R. Hoar, for the plaintiff; G. F. Farley and George Bancroft (of Suffolk), for the defendant.

It is obvious to remark, that the foregoing case is one of the most extraordinary character, and *interesting*, if not alarming to the whole profession; but as it will probably be again submitted to a jury, propriety forbids any comment upon its merits.

*The use of Iron as a Prophylactic against Cholera.*—I wish to suggest to those exposed to the influence of cholera, the internal use of iron as a prophylactic.

I conjecture that when the blood is well impregnated with iron, it is rendered less prone to undergo the morbid change in which many epidemic diseases primarily consist. The experience of an individual is insufficient to put this conjecture to the test; and as regards cholera, I have not even that experience to offer. During the prevalence of the Irish fever, I believe I did obtain a little negative evidence in support of my opinion, but not nearly sufficient to establish it.

Taken in the form of pill along with solid food, iron scarcely ever disagrees, provided neither fever nor active inflammation be present. Any one disposed to try it against the contagion—for such I believe it—of cholera, will find a grain or two of the sulphate, made into a pill, with extract of gentian, to be taken during, or immediately after, each of the principal meals, a convenient method.—M. D.—*Lancet.*

